# Effects of perioperative anesthetics on the postoperative prognosis of patients undergoing surgery for cervical cancer

**DOI:** 10.3389/fphar.2025.1536663

**Published:** 2025-03-10

**Authors:** Linyan Deng, Tingting Wang, Qiaofeng Zhang, Shaofang Shu, Xiangdong Chen

**Affiliations:** ^1^ Department of Anesthesiology, Union Hospital, Tongji Medical College, Huazhong University of Science and Technology, Wuhan, China; ^2^ Institute of Anesthesia and Critical Care Medicine, Union Hospital, Tongji Medical College, Huazhong University of Science and Technology, Wuhan, China; ^3^ Key Laboratory of Anesthesiology and Resuscitation (Huazhong University of Science and Technology), Ministry of Education, Wuhan, China

**Keywords:** cervical cancer, anesthetics, perioperative, metastasis, recurrence

## Abstract

Cervical cancer is a common malignancy among women, and tumor excision is the most common surgical intervention. Anesthetics used during surgery include general intravenous, volatile, local anesthetics, sedative and analgesic. Studies have shown that the selection of perioperative surgical methods and anesthetics may influence postoperative metastasis and cancer recurrence through their effects on the immune response and tumor cells. Therefore, the selection of perioperative anesthetic has a significant impact on patients undergoing surgery for cervical cancer. This study summarizes the effects and related mechanisms of common anesthetics on the prognosis of patients undergoing surgery for cervical cancer to provide a basis for developing more optimal anesthesia protocols.

## 1 Introduction

Cervical cancer is the fourth most common malignancy in women worldwide (Bray et al., 2024), and surgical resection is the primary intervention. However, the postoperative recurrence rate of cervical cancer is still very high. Patients undergoing surgery for cancer may experience tumor cell spread to the circulating blood or lymphatic system, or may shed tumor cells during resection. These factors affect postoperative metastasis and the recurrence of cervical cancer. Studies have shown that the selection of perioperative anesthetic has an impact on postoperative recurrence, metastasis, and immune function (Kim, 2018; Ackerman et al., 2021).

Anesthetics include general intravenous, volatile, local anesthetics, sedative and analgesic. Propofol, a widely used intravenous anesthetic, may exert antitumor effects by modulating noncoding RNAs, signaling pathways, and host immune functions (Liu et al., 2021; Liu et al., 2016). There is evidence that volatile anesthetics may also influence cancer recurrence and metastasis by modulating immunity (Wang et al., 2022; Buckley et al., 2014). Long-term exposure with sevoflurane negatively affects estrogen and progesterone regulation (Dogru et al., 2017), and estrogen and progesterone may influence anesthetic dose (Shimizu et al., 2010; Basaran et al., 2019). In addition, studies have shown that estrogen may promote the development of cervical cancer (Baik et al., 2022), while progesterone often inhibits the progression of the disease (Yoo et al., 2013). Nonsteroidal anti-inflammatory drugs (NSAIDs) may have an antitumor effect by exerting anti-inflammatory effects (Saka-Herrán et al., 2021). In addition, local anesthetics may inhibit tumor progression by regulating immunity and inducing apoptosis (Pérez-González et al., 2017; Chen et al., 2022). These findings suggest that perioperative anesthetics may have varying effects on cervical cancer. Therefore, anesthesiologists can improve the prognosis of patients undergoing surgery for cervical cancer by selecting the appropriate anesthetic agents.

The present article reviews the postoperative effects of intravenous, volatile, local anesthetics, sedative and analgesic on patients undergoing surgery for cervical cancer and their underlying mechanisms (Table1).

**TABLE 1:** Effects and related mechanisms of anesthetics on cervical cancer cells.

Agents	Cell lines	Study type	Treatment	Mechanisms	Functions				References
					Proliferation	Apoptosis	Migration	Invasion	
Propofol	Caski and SiHa	*In vitro*/*in vivo*	50 μM,24 h; 20 mg/kg, once a week for 5 weeks	miR155HG↓	↓	-	-	↓	Du et al. (2021b)
	HeLa, Caski and C33A	*In vitro*/*in vivo*	10 μg/mL, 24 h; 50 mg/kg, once a day for 3 weeks	HOTAIR↓→mTOR/p70S6K↓	↓	↑	-	-	Zhang et al. (2015)
	HeLa and SiHa	*In vitro*/*in vivo*	10 μg/mL, 24 h; 35 mg/kg, once a week for 4 weeks	HOTAIR↓→miR-129-5p/RPL14↑	↓	↑	↓	↓	Sun et al. (2021)
	HeLa	*In vitro*/*in vivo*	0, 2.5, 5, 10 mug/mL 10, 20, 50 mg/kg	wnt/beta-catenin↓	↓	↑	↓	↓	Huang et al. (2020b)
	MS751, Caski, SiHa, Hela and C33A	*In vitro*	10–100 μmol/L,72 h	EGFR/JAK2/STAT3↑	↓	↑	-	-	Li et al. (2017)
	C33A and HeLa	*In vitro*	1–20 (10) μg/mL,24 h	SLC7A11/GPX4↓, Ubiquinol/CoQ10/FSP1↓, YAP/ACSL4/TFRC↑→Paclitaxel-Initiated Cell Ferroptosis↑	↓	↑	-	-	Zhao et al. (2022)
	HeLa	*In vitro*	400 μM, 24 h	AMPK/mTOR↑, ER stress↑→ impair autophagic flux	↓	↑	-	-	Chen et al. (2018)
Dexmedetomidine	HeLa and SiHa	*In vitro*	-	JAK/STAT↓	↓	-	↓	↓	Xu et al. (2021)
Sevoflurane	HeLa SiHa and C33A	*In vitro*	2%, 4%, 8%, 6 h	RhoA/MYPT1/MLC↓; Ras/ERK/AKT↓	↓	-	↓	↓	Ding et al. (2019)
	HeLa	*In vitro*	-	miR-203↑	↓	↑	↓	↓	Zhang et al. (2020b)
	HeLa and SiHa	*In vitro*	3%, 2 h	-	↑	↓	↑	-	Xue et al. (2019)
	Caski and HeLa	*In vitro*	1%, 2%, 3%, 2h/4 h	PI3K/AKT↑, ERK1/2↑→HDAC6↑	↑	-	↑	↑	Zhang et al. (2020a)
Isoflurane	SiHa and Caski	*In vitro*	1%, 2%, 3%, 2h/4 h	p-mTOR↑→HDAC6↑	↑	-	-	-	Zhang et al. (2021)
	Cervical cancer cells	*In vitro*	-	miR-375↓	↑	↓	-	↑	Li et al. (2019)
	HeLa	*In vitro*/*in vivo*	-	AMPK/mTOR↑→autophagy↑	↓	↑	-	-	Wei et al. (2021)
	HeLa	*In vitro*/*in vivo*	1.4%, 6 h; 1.4%, 2 h, once a day for 10 days	-	↓	↑	-	-	Ma et al. (2020)
Morphine	C33A and Caski	*In vitro*	0.25–4 μM, 24 h	EGFR↑; RhoA↑	↑	-	↑	-	Yu et al. (2022)
Sufentanil	HeLa	*In vitro*	500 nmol/L	PI3K/AKT/mTOR↓	↓	↑	-	-	Jin, Sun, and Liu, (2021)
Celecoxib	HeLa	*In vitro*	5–160 μmol/L, 24–96 h	-	↓	-	-	-	Wang et al. (2012)
	HeLa	*In vitro*	24 h	tNOX↓	↓	-	-	-	Morre and Morre, (2006)
	HeLa, Caski and C33A	*In vitro*	50 μM, 8 h	caspase-8↑; NF-κB↓	↓	↑	-	-	Kim et al. (2004)
	HeLa, Caski and C33A	*In vitro*	50 μM, 8 h	GADD153↓	-	↑	-	-	Kim et al. (2007)
	HeLa	*In vitro*	100 μM, 24 h	survivin↓	-	↑	-	-	Fukada et al. (2007)
	HeLa	*In vitro*	40 μM, 12/24 h	P53↑	↓	↑	-	-	Setiawati and Setiawati, (2016)
Lidocaine	HeLa	*In vitro*	1 mM, 3 mM, 48 h	Ki67↓, and change distribution of Ki-67	↓	-	-	-	Haraguchi-Suzuki et al. (2022)
	HeLa	*In vitro*	500 μM, 24 h	lncRNA-MEG3↑→miR-421↓→BTG1↑→PI3K/AKT↓	↓	↑	-	-	Zhu and Han, (2019)
Ropivacaine	SiHa and Caski	*In vitro*	0.25, 0.5.1 mM, 72 h	miR-96↓→MEG2↑→pSTAT3↓	↓	↑	-	-	Chen et al. (2020)

-, indeterminate or limited data; ↑, increase; ↓, decrease.

## 2 Intravenous anesthetic agents

### 2.1 Propofol

Available data suggest that propofol can inhibit proliferation and metastasis, and induce apoptosis of malignant tumors (Yu et al., 2020; Liu et al., 2021; Du et al., 2021a; Hu et al., 2022), thereby reducing the risk for cancer recurrence and improving survival, especially in patients undergoing major surgery for cancer (Yap et al., 2019; Sessler and Riedel, 2019). Propofol may affect the development and prognosis of cancer by regulating microRNAs (miRNAs), and modulating signaling pathways and host immune function.

miRNA dysregulation is associated with cancer progression. An *in vitro* study demonstrated that propofol inhibits the growth and invasion of cervical cancer cells and epithelial-mesenchymal transition by inhibiting miR155HG (Du Y. et al., 2021). The long noncoding RNA (lncRNA) Hox transcript antisense intergenic RNA (HOTAIR) has been reported to be associated with tumor recurrence in cervical cancer (Li et al., 2015; Zhou et al., 2020). Propofol inhibits activation of the mammalian target of rapamycin (mTOR)/p70S6K pathway (Zhang et al., 2015) and modulates the HOTAIR/miR-129-5p/ribosomal protein L14 (RPL14) axis (Sun et al., 2021) by decreasing HOTAIR expression, which subsequently inhibits the growth of cervical cancer cells.

Signaling pathways play an important role in regulating the development and progression of cancer. Researchers have found that the overexpression of epidermal growth factor receptor (EGFR) and signal transducer and activator of transcription (STAT3) are associated with poor prognosis in patients with cervical cancer, and propofol can enhance the antitumor effect of cisplatin on cervical cancer cells by inhibiting the EGFR/Janus tyrosine kinase 2 (JAK2)/STAT3 pathway (Li et al., 2017).In addition, it has been reported that propofol induces ferroptosis by regulating the solute carrier family 7 member 11 (SLC7A11)/glutathione peroxidase 4 (GPX4), ubiquinol/CoQ10/FSP1, and Yes1 associated transcriptional regulator (YAP)/acyl-CoA synthetase long-chain family member 4 (ACSL4)/transferrin receptor (TFRC) pathways, and has a synergistic antitumor effect with paclitaxel (Zhao et al., 2022). Propofol also induces endoplasmic reticulum stress, regulates adenosine monophosphate-activated protein kinase (AMPK)/mTOR signaling, and impairs autophagic flux, thereby inhibiting the growth of HeLa cells (Chen et al., 2018). In addition, inhibition of the Wnt/β-catenin pathway may be the underlying mechanism by which propofol inhibits the growth and migration of cervical cancer cells (Huang et al., 2020a).

Atomic force microscopy has been used to analyze propofol-induced changes in the ultrastructure of the cell membrane of cervical cancer cells. Cervical cancer cells exhibit an evident decrease in membrane roughness, and their lamellipodia significantly retract or disappear after treatment with propofol, which affects cell migration (Zhang et al., 2016).

The antitumor effects of propofol have been verified in animal trials. By generating xenograft tumor and lung metastasis model animals, researchers have found that propofol significantly reduced the level of MIR155HG in tumor tissues and suppressed tumor growth and metastasis of cervical carcinoma cells *in vivo* (Du et al., 2021a). Additionally, propofol inhibited increases in tumor size in a cervical cancer xenograft model (Zhang et al., 2015; Huang et al., 2020a; Sun et al., 2021).

Patients who experience recurrence and metastasis are usually due to immunosuppression. Natural killer (NK) cells play an important role in controlling tumor metastasis (López-Soto et al., 2017; Lo et al., 2020). A clinical study found that, during the perioperative period in laparoscopic radical procedures in patients with cervical cancer, the number of CD3-positive (+), CD4^+^, and NK cells, and the ratio of CD4+/CD8+ cells in the sevoflurane anesthesia group were significantly lower than those in the propofol anesthesia group, indicating that propofol may be beneficial in reducing perioperative immunosuppression, thus mitigating adverse prognoses in patients with cervical cancer (Liu et al., 2016). In addition, a retrospective study comparing the effects of anesthesia on overall, cancer-specific, and recurrence-free survival in patients undergoing surgery for cervical cancer reported that propofol was associated with better long-term outcomes (Takeyama et al., 2021). These findings suggest that propofol has an antitumor effect on cervical cancer.

### 2.2 Ketamine

Ketamine acts rapidly and does not cause respiratory depression, which supports its safety and widespread use. However, studies have demonstrated that ketamine has immunosuppressive effects that may adversely affect tumor prognosis. Ketamine may promote tumor metastasis by inhibiting NK cell activity and increasing the number of regulatory T cells (Tregs) to suppress immunity (Melamed et al., 2003; Hou et al., 2016). However, recent clinical and *in vitro* studies have shown that ketamine has no effect on the cytotoxicity of NK cells in patients undergoing surgery for cancer (Kubota et al., 2022; Cho et al., 2021). Ketamine can even inhibit the proliferation and migration of cancer cells and induce ferroptosis (Li D. et al., 2021; Hu et al., 2020). Two clinical studies found that ketamine inhibited immune function in cervical cancer patients undergoing surgery (Wang et al., 2011; Jiang et al., 2022). Studies investigating the role of ketamine in cervical cancer cells are lacking and, thus, merit elaboration.

### 2.3 Etomidate

Etomidate is beneficial for improving hemodynamic stability during surgery. Many *in vitro* studies have shown that etomidate plays an antitumor role by suppressing cell proliferation and migration and promoting cell apoptosis (Chu et al., 2019; Li H. et al., 2021). In addition, a retrospective cohort study found that etomidate was more detrimental than propofol to the prognosis of patients who underwent radical gastrectomy (Lu et al., 2022). However, research investigating the relationship between etomidate and the progression of cervical cancer is lacking and, as such, merits further promotion.

### 2.4 Dexmedetomidine

Dexmedetomidine (DEX) is an α-2A adrenergic receptor (ADRA2A) agonist with unique sedative and analgesic effects and minimal respiratory depression, and is widely used in the clinic. A meta-analysis revealed that DEX can reduce perioperative stress and inflammation, protect immune function in patients undergoing surgery, reduce postoperative complications, and improve prognosis (Wang et al., 2019). Studies have found that DEX promotes the proliferation and metastasis of breast, lung and colon cancers (Lavon et al., 2018), but plays an antitumor role in ovarian and esophageal cancers (Shin et al., 2021; Hu et al., 2021). These conflicting results may be attributed to differences in the duration and concentration of DEX exposure and tumor models. DEX plays multiple roles in cancer cells, the effects on tumor progression are complex and non-uniform, and there is a lack of high-quality clinical evidence on the prognostic effects of DEX in cancer patients. *In vitro* studies have shown that DEX inhibits cervical cancer cell proliferation and migration through the JAK/STAT signaling pathway (Xu et al., 2021). Data regarding the effects of DEX on the progression of cervical cancer are insufficient and further research is needed.

In summary, propofol and DEX may exert antitumor effects on cervical cancer, whereas the effects of ketamine and etomidate remain uncertain. More studies are needed to explore the relationship between intravenous anesthetics and the progression of cervical cancer and the molecular mechanisms involved, especially in clinical trials, to verify the effect of intravenous anesthetics on the long-term prognosis of cervical cancer in humans.

## 3 Volatile anesthetic agents

### 3.1 Sevoflurane

**FIGURE 1:**
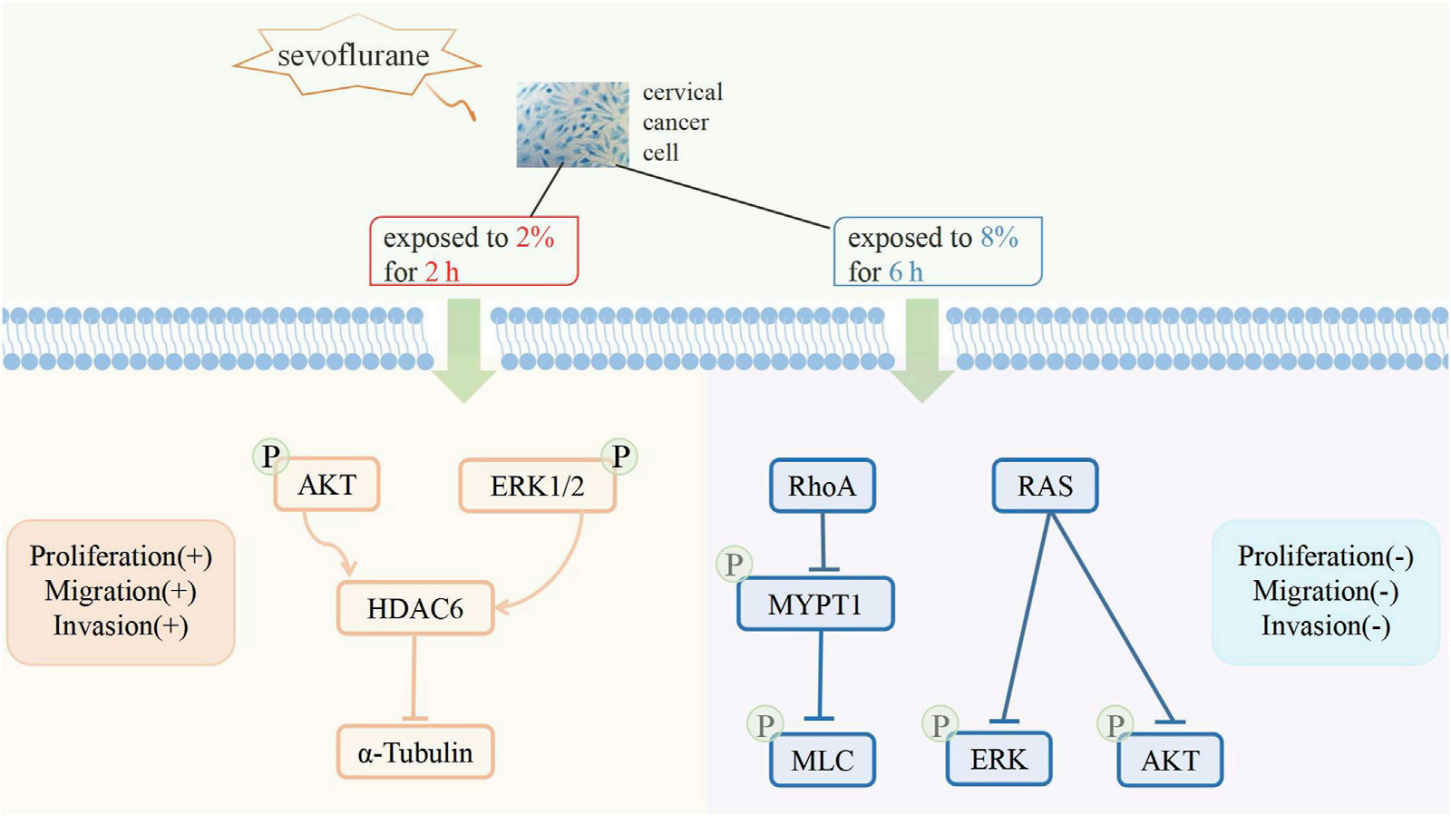
Different effects of sevoflurane on cervical cancer cells.

The biological effect of sevoflurane on cancer cells remains unclear. Sevoflurane enhances the proliferation and migration of ovarian cancer cells. Sevoflurane enhances the proliferation and migration of ovarian cancer cells (Ishikawa et al., 2021; Hu et al., 2022), but inhibits proliferation and migration of colorectal cancer and gastric cancer cells (Fan et al., 2019; Yong et al., 2021). Studies have shown that sevoflurane mediates antiproliferation and antimigration of cervical cancer cells by targeting resistance to audiogenic seizures (Ras) and ras homolog family member A (RhoA) and up-regulating miR-203 (Ding et al., 2019; Zhang et al., 2020a). However, another study suggested that sevoflurane promotes the proliferation of cervical cancer cells but has no effect on cisplatin sensitivity (Xue et al., 2019). Exposure to clinically relevant concentrations of sevoflurane induces the upregulation of histone deacetylase 6 through the phosphatidyl inositol 3-kinase (PI3K)/protein kinase B (AKT-) and extracellular regulated protein kinases (ERK1/2-) signaling pathways, thereby promoting the proliferation and metastatic potential of cervical cancer cells (Zhang et al., 2020a). These conflicting results can be attributed to the duration and concentration of sevoflurane exposure (Figure 1).

A retrospective cohort study showed that the prognosis of patients undergoing surgery for cervical cancer and received sevoflurane anesthesia was worse than that of those who received propofol anesthesia (Takeyama et al., 2021). However, studies have shown that the anesthetic effect of sevoflurane combined with remifentanil in laparoscopic radical surgery for cervical cancer is better than that of propofol combined with remifentanil. Sevoflurane combined with remifentanil anesthesia can also improve perioperative cellular immune function and relieve physiological stress (Wang et al., 2022; Suo et al., 2022).

### 3.2 Isoflurane

Isoflurane promotes proliferation, migration, and invasion in most cancer cells (Zheng et al., 2022; Benzonana et al., 2013). Isoflurane upregulated the expression of histone deacetylase 6, which is associated with the mTOR-dependent pathway, thereby promoting the proliferation of squamous cervical cancer cells (Zhang et al., 2021). Isoflurane promoted cell proliferation and inhibited apoptosis in cervical cancer by downregulating miR-375 (Li et al., 2019). However, another study reported that isoflurane inhibited the proliferation of cancer cells and promoted apoptosis and autophagy through the AMPK/mTOR pathway (Wei et al., 2021). In addition, isoflurane has a potential inhibitory effect on cervical cancer in nude mice (Ma et al., 2020). To our knowledge, no laboratory studies have investigated the relationship between isoflurane and the progression of cervical cancer.

### 3.3 Desflurane

Conclusions regarding the effects of desflurane on cancer prognosis are mixed. Studies have suggested that total intravenous and desflurane anesthesia have comparable effects on cancer prognosis in patients undergoing surgery (Cho et al., 2021). Studies have also shown that the survival rate of patients with gastric and colon cancer undergoing surgery and received total intravenous anesthesia with propofol was higher than that of those who received desflurane anesthesia during the 3–5-year follow-up period after surgery (Wu et al., 2018; Huang et al., 2020b). There have been no laboratory or clinical studies investigating the relationship between desflurane and the progression of cervical cancer.

Volatile anesthetics can affect cancer recurrence and metastasis by altering innate immunity and decreasing NK cell cytotoxicity (Buckley et al., 2014). One study demonstrated that propofol was superior to sevoflurane in protecting circulating lymphocytes in patients undergoing laparoscopic radical hysterectomy for cervical cancer (Liu et al., 2016). Moreover, the effect of volatile anesthetics on the prognosis of patients with cervical cancer remains controversial; as such, further laboratory and clinical trials are required to obtain more conclusive results.

## 4 Sedative and analgesic agents

### 4.1 Opioids

Opioids are the most commonly used perioperative analgesics in patients with cancer who undergo surgery. Opioids may contribute to cancer progression by suppressing immunity and promoting cancer-related inflammation, tumor cell migration, and angiogenesis (Perry and Douglas, 2019; Amaram-Davila et al., 2020; Abdel Shaheed et al., 2022). However, studies have shown that opioids may play an antitumor role by activating tumor cell apoptosis and reducing invasion and metastasis (Connolly and Buggy, 2016; Lec et al., 2020; Ramirez et al., 2021). These conflicting results may be related to the type and dosage of opioids, and the type of cancer.

Morphine is an opioid-based analgesic. *In vitro* studies have shown that morphine can promote the proliferation of cervical cancer cells by activating the opioid receptor-dependent EGFR-mediated signaling pathway and stimulate migration by activating the opioid receptor-independent RhoA-mediated signaling pathway (Yu et al., 2022). In contrast, sufentanil induces apoptosis in cervical cancer cells through the PI3K/AKT/mTOR signaling pathway (Jin et al., 2021). Another study reported that morphine suppresses immunity through the JAK3/STAT5 pathway (Jiang et al., 2022). A clinical study reported that bupivacaine combined with morphine inhibited postoperative immune function in patients with cervical cancer, whereas ropivacaine combined with fentanyl had a lower immunosuppressive effect (Dianqing et al., 2007). In addition, compared with fentanyl, remifentanil had a minimal effect on T lymphocytes after surgery for cervical cancer (Lu et al., 2018). The effect of opioids on the long-term prognosis of patients undergoing surgery for cervical cancer has not been extensively studied, and further research, especially clinical studies, is required to confirm this finding.

### 4.2 Benzodiazepines

Benzodiazepines have both sedative and hypnotic effects. The results of two meta-analyses suggested that there may be no association between benzodiazepines and survival in patients with cancer (O'Donnell et al., 2019; O'Donnell et al., 2018). However, *in vitro* and animal studies have shown that diazepam and midazolam exhibit antitumor and anti-inflammatory effects (Oshima et al., 2022; Wang et al., 2018; Kim et al., 2008). Midazolam may exert its antitumor effect by inhibiting the local invasion of tumor-associated neutrophils and tumor-associated macrophages, or by inhibiting their proliferation and migration. However, no laboratory or clinical studies have investigated the relationship between benzodiazepines and the progression of cervical cancer; therefore, further research is needed.

### 4.3 NSAIDs

NSAIDs are commonly used as analgesics during surgery. It is well known that inflammatory responses to tumor tissues may promote the occurrence and development of tumors (Nasry et al., 2018; Balkwill and Mantovani, 2001). In contrast, NSAIDs play an anti-inflammatory role by inhibiting cyclooxygenase (COX) enzymes to inhibit the synthesis of prostaglandins, thus playing an antitumor role (Retsky et al., 2013; Brusselaers and Lagergren, 2018).

NSAIDs have been shown to exert antitumor effects in cervical cancer *in vitro* and in animal models by inhibiting growth and migration, and inducing apoptosis (Soriano-Hernandez et al., 2015; Marinov et al., 2021; Kim et al., 2003). Celecoxib radio-sensitizes HeLa cells by downregulation of COX-2 and vascular endothelial growth factor C (VEGF-C) (Wang et al., 2012). Moreover, in addition to COX-2, NSAIDs, such as celecoxib, inhibit HeLa growth through tNOX, a cancer-specific cell surface oxidase (ECTO-NOX), via protein disulfide-thiol interchange activity (Morre and Morre, 2006). *In vitro* studies have also shown that NF-kappaB, DNA damage inducible gene (GADD153) and survivin play important roles in celecoxib-induced apoptosis (Kim et al., 2004; Kim et al., 2007; Fukada et al., 2007). Moreover, celecoxib induces apoptosis and cell cycle arrest of cervical cancer cells by upregulation of p53 through various molecular mechanisms (Setiawati and Setiawati, 2016; Saha et al., 2012). However, a meta-analysis revealed that the long-term use of NSAIDs was not associated with the progression of cervical intraepithelial neoplasia (Grabosch et al., 2018; Grabosch et al., 2014). More clinical trials are needed to confirm the relationship between NSAIDs and the long-term prognosis of cervical cancer.

## 5 Local anesthetic agents

Local anesthetics are typically used as regional and neuraxial anesthetics during the perioperative period. Studies have shown that local anesthetics can enhance immune response and reduce inflammation, thus exhibiting antitumor effects (Pérez-González et al., 2017; Ramirez et al., 2015). In addition, many *in vitro* experiments have demonstrated that local anesthetics may inhibit the growth, migration, and invasive capacity of cancer cells, and induce apoptosis and autophagy through various mechanisms, thereby playing an inhibitory role in tumor progression (Chen et al., 2022; Li et al., 2018). However, a meta-analysis revealed that perioperative local anesthesia may improve the survival of patients with cancer after oncological surgery, although there is no evidence supporting a correlation with cancer recurrence after oncological surgery (Sun et al., 2015).

Studies have found that lidocaine, a commonly used local anesthetic, decreases Ki-67 expression in cervical cancer cells, thus inhibiting their growth (Haraguchi-Suzuki et al., 2022). LncRNA maternally expressed gene 3 (lncRNA-MEG3) is associated with the progression of cervical cancer (Wang et al., 2017). In an *in vitro* study, lidocaine inhibited the proliferation and induced apoptosis of cervical cancer cells by activating the lncRNA-MEG3/miR-421/BTG anti-proliferation factor 1 (BTG1) pathway (Zhu and Han, 2019). Ropivacaine has been shown to exert similar antitumor effects by reducing miR-96 expression and upregulating MEG2 expression, leading to STAT3 dephosphorylation (Chen et al., 2020). Clinical studies have demonstrated that intraoperative lidocaine has a protective effect on perioperative immune function in patients with cervical cancer undergoing radical hysterectomy, which may inhibit tumor metastasis (Wang et al., 2015; Hong and Lim, 2008). Clinical studies investigating the relationship between local anesthetics and the progression of cervical cancer are lacking. As such, further research is needed to determine the long-term effects of local anesthetics in patients undergoing surgery for cervical cancer.

## 6 Conclusion

**FIGURE 2:**
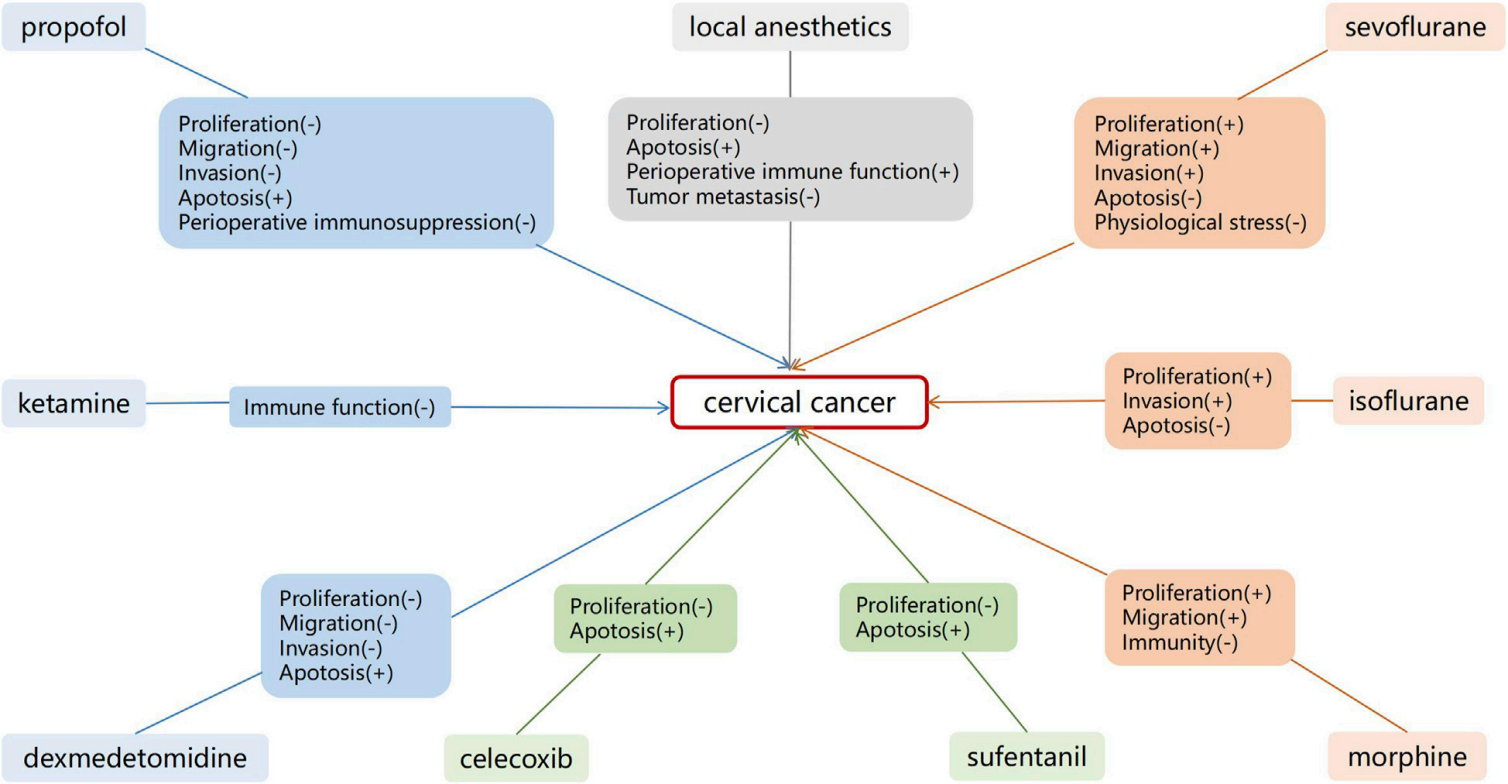
Effects of anesthetics on cervical cancer cells. Shorter duration of sevoflurane and isoflurane use may have a tumor promoting effect on cervical cancer.

Perioperative anesthetics affect cancer progression. Herein, we summarized the long-term postoperative effects of anesthetics on patients with cervical cancer. Current *in vitro* studies have demonstrated that propofol, DEX, remifentanil, celecoxib, and local anesthetics, such as lidocaine, may exert antitumor effects on cervical cancer. The effects of sevoflurane and isoflurane on cervical cancer, however, remain controversial (Figure 2). Studies have demonstrated that shorter periods of sevoflurane and isoflurane use may have tumor-promoting effects on cervical cancer, although further basic research is needed to confirm this.

Clinical trials aimed at confirming that the perioperative use of DEX, remifentanil, and celecoxib can reduce tumor recurrence and prolong the survival of patients undergoing surgery for cervical cancer are lacking. Furthermore, randomized controlled trials that evaluate the impact of perioperative lidocaine use on patients undergoing cervical cancer surgery have not included assessments of patient survival (Wang et al., 2015; Hong and Lim, 2008). The investigation into the effects of perioperative sevoflurane and propofol on the prognosis of patients with cervical cancer is limited to retrospective cohort studies (Takeyama et al., 2021), with a distinct lack of randomized controlled trials. Presently, there is an absence of robust clinical evidence, such as randomized controlled trials or high-quality meta-analyses, to substantiate the influence of various anesthetic agents on the long-term prognosis of patients following cervical cancer surgery. Therefore, more prospective studies and long-term follow-ups of cancer recurrence after surgery are needed to develop more sophisticated and effective anesthesia protocols for patients undergoing surgery for cervical cancer.
